# Silver-Neodymium Codoped Lithium Aluminum Metaphosphate Glasses for Radio-Photoluminescence Dosimeter

**DOI:** 10.3390/ma15165527

**Published:** 2022-08-11

**Authors:** Xiben Ma, Jimeng Cheng, Sijun Fan, Xin Wang, Wei Chen, Shubin Chen, Lili Hu

**Affiliations:** 1 Key Laboratory of Materials for High Power Laser, Shanghai Institute of Optics and Fine Mechanics, Chinese Academy of Sciences, Shanghai 201800, China; 2 Center of Materials Science and Optoelectronics Engineering, University of Chinese Academy of Sciences, Beijing 100049, China

**Keywords:** Ag–Nd-codoped phosphate glass, energy transfer, fluorescence, radiation dose, radio-photoluminescence (RPL) glass dosimeters

## Abstract

Commercial radio-photoluminescence (RPL) glass dosimeters generally use Ag single-doped phosphate glass as a single-wavelength sensor. Now, a novel type of Ag–Nd-codoped phosphate glass has been developed, which can be applied to dual-wavelength or multi-wavelength RPL sensors, and can thus improve the accuracy and stability of RPL dosimeters. An anhydrous 99.5 (0.7LiPO_3_–0.3Al (PO_3_)_3_) −0.25Ag_2_O–0.25Nd_2_O_3_ glass was prepared and irradiated at different doses, and then the absorption, fluorescence, infrared transmission spectra, as well as fluorescence lifetimes were tested and analyzed. The results show that there is an energy transfer between the Ag defect center and Nd^3+^ ions, and the transfer efficiency using 380 nm excitation is greater than that using 310 nm excitation. Aside from the 650 nm fluorescence of the Ag defect center, strong 882 nm and 1054 nm fluorescences of Nd ions are exhibited. It is possible that these fluorescences would allow the developed Ag–Nd-codoped phosphate glass to be applied to new RPL glass sensors and dosimeters.

## 1. Introduction

Radio-photoluminescence (RPL) is a form of photoluminescence in which the luminescence center is a defect produced by irradiation. Since the fluorescence intensity is proportional to the radiation dose, RPL is widely used in dosimetry [1,2,3,4]. Dosimetry is the measurement of a dose with a dosimeter. RPL glass dosimeters exhibit high sensitivity, small energy dependence, good dose linearity, and are able to make repeated readings [5,6,7]. RPL glass dosimeters are widely used in the nuclear power industry, as well as for environmental dose monitoring and personal dose monitoring. The Ag-doped phosphate RPL glass dosimeter was first invented by Schulman et al. in 1951 [8]. In the 1960s, Yokota et al. [9,10] optimized the composition and fabrication method for the glass, which greatly improved the RPL performance of the glasses. However, there were still a few flaws, such as the photoluminescence (PL) generated by dirt on the glass surface and on the dirt itself. With the application of pulsed ultraviolet (UV) light and UV LED to the measuring devices [11], the RPL glass dosimeters eliminated the PL noise and dominated the market. After continuous development, Ag-doped phosphate glasses as RPL dosimeters have been researched and commercialized by Chiyoda Technol Corporation [12].

Irradiated Ag-doped phosphate glasses generate stable luminescence centers, and the luminescence mechanism of Ag defect centers in RPL glass dosimeters has been discussed by many scholars [13,14,15,16,17]. Electron and hole centers are formed when phosphate glasses are exposed to ionizing radiation. A portion of the Ag^+^ ions change to Ag^0^ by capturing an electron (Ag^+^ + e^−^ → Ag^0^), and a portion of the Ag^+^ ions become stable Ag^2+^ by capturing a hole (Ag^+^ + h^+^ → Ag^2+^). Ag defect centers will emit fluorescence at 600–700 nm when excited by pulsed UV light, which is called RPL. Due to the hole transfer, the RPL intensity continues to increase after irradiation stops and reaches saturation at room temperature, which is termed the build-up effect. The current commercial RPL glass dosimeters are made of Ag-doped alkali aluminum metaphosphate glasses. The lithium aluminum metaphosphate glass system is characterized by a small ionic radius, small effective atomic number, and excellent UV transmission properties, all of which are beneficial to reducing the energy dependence of the dosimeter and improving the chemical stability of the phosphate glasses [10]. Fan et al. [18,19] pointed out that the RPL intensity is related to the content of alkali metals in the glasses, and the higher the content of alkali metals, the better the ion diffusion and the trapping of hole defects by Ag^+^. A large quantity of Al_2_O_3_ will affect the capture of hole defects by Ag^+^ and the formation of luminescence centers, even though it can strengthen the structure of phosphate glasses and improve chemical stability [20,21,22]. Tomoaki Kuro et al. [23] systematically discussed the photoluminescence (PL), scintillation, and dosimeter features of rare-earth ion (RE)-doped NaPO_3_–Al (PO_3_)_3_ glasses. It was discovered that Nd- and Tb-doped glasses showed high response and stability when tested for the dose-response of thermally stimulated luminescence (TSL) of rare-earth ions in the dosage range of 10 mGy–10 Gy. However, related research examining the relationship between the fluorescence intensity of Ag–Re-codoped phosphate glasses and the size of the radiation dose administered has not been reported by scholars.

Based on the above discussion, this paper aims to develop a new type of Ag–Nd-codoped phosphate glass material to expand the research of Ag-doped phosphate RPL glass dosimeters. Ag and other rare earth-doped [24,25,26,27] dosimeter glasses are also desired for further investigation. In this work, the matrix, Nd-doped, Ag-doped, and Ag–Nd-codoped LiPO_3_–Al (PO_3_)_3_ glasses were prepared. And the absorption, fluorescence, transmission spectra, and fluorescence lifetime of Ag defect centers were measured. The RPL intensity, the linear relationship between the fluorescence intensity of RPL and Nd^3+^ ions and the radiation dose, and the energy transfer efficiency from the Ag defects center to Nd^3+^ ions were systematically investigated at 310 and 380 nm excitation.

## 2. Experiment Details

### 2.1. Sample Preparation

The matrix, Nd-doped, Ag-doped, and Ag–Nd-codoped phosphate glasses were prepared by a high-temperature melting and quenching method. The compositions of the glass sample are listed in Table 1. The starting materials were anhydrous reagent powders of LiPO_3_, Al (PO_3_)_3_, Ag_2_O, and Nd_2_O_3_. The 200 g raw materials were fully mixed and poured into the preheated corundum crucible. Then, the corundum crucible was transferred to a silicon carbide rod electric furnace at a temperature of 1100 °C, where it remained for 60 min. After dehydration with CCl_4_ and clarification at a high temperature, the glass liquid was poured into a preheated steel mold. Finally, the formed glass was sent to an annealing furnace at 430 °C for 24 h.

### 2.2. Characterization of the Bulk Glass

The annealed glasses were processed into 10 mm × 10 mm × 2 mm thin sheets and polished on both sides as test samples. The irradiation source was an X-RAD 160 (Precision X-Ray Inc., Faxitron, Tucson, AZ, USA), which used Alanine Dosing Tablets (E2044562) to calibrate the ^60^Co gamma total dose. The dose calibrated by the instrument was also verified by an X-ray dosimeter. The X-ray energy of the X-RAD 160 was 0.16 MeV. The target material was a tungsten target, the working voltage was 160 kV, and the window material was a beryllium window. The product of dose rate and time is the radiation dose, and the dose rate was 24 Gy/min. The radiation doses of each group of samples were 0, 50, 100, 200, and 250 Gy, respectively. All samples with the same dose were irradiated at the same position to prevent radiation errors.

The absorption spectrum was measured with the Lambda 950UV/VIS/NIR spectrophotometer (Perkin–Elmer, Waltham, MA, USA), with a test range of 200–600 nm and a scan step of 1 nm. The Edinburgh Instrument FLSP920 steady-state/transient fluorescence spectrometer was the test instrument for the fluorescence spectrum and radiation lifetime. The detector models used in the fluorescence test were R928 UV visible PMT (band range 350–800 nm) of the Hamamatsu company in Japan and 316ldc-dd CCD detector (band range 500–1100 nm) of the Andor company in the UK, respectively. The sensitivity calibration curve file was included in test software. For each measurement of the sample, a calibration curve will be selected to correct the results to ensure accurate test results. The fluorescence spectra were tested in the range of 400–800 nm and 500–1100 nm, using a Xe lamp as the light source, while the lifetime of the Ag defect centers was measured using a hydrogen lamp as the light source. All spectral tests were performed at room temperature.

## 3. Results and Discussion

### 3.1. The Absorption Spectra

**Figure 1:**
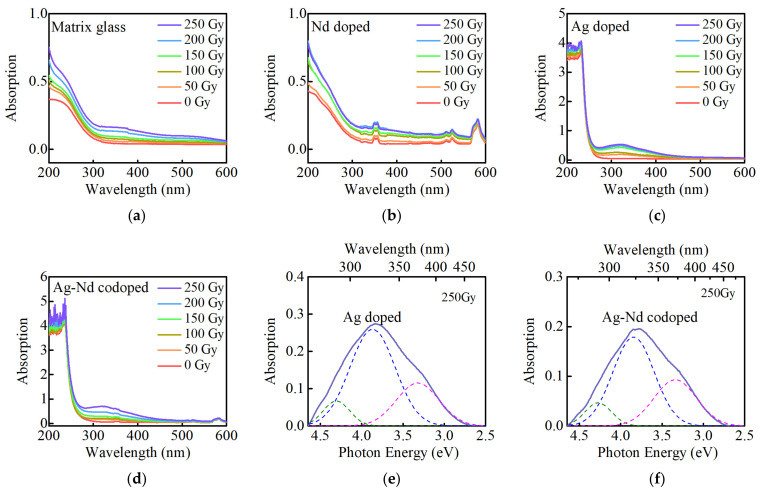
(**a**) The Absorption spectra of the matrix; (**b**) Nd-doped; (**c**) Ag-doped; (**d**) Ag–Nd-codoped glasses at various radiation doses; (**e**) Gaussian peaks for the induced absorption spectra of Ag-doped glass at 250 Gy; (**f**) Gaussian peaks for the induced absorption spectra of Ag–Nd-codoped glass at 250 Gy.

The absorption spectra of the matrix, Nd-doped, Ag-doped, and Ag–Nd-codoped phosphate glasses at various radiation doses are presented in Figure 1. The irradiated matrix glasses produce absorption in the range of 250–600 nm, and the absorption intensity improves with increasing radiation dose, as shown in Figure 1a. The absorption band peaking at 520 nm is the defect absorption caused by the capture of a hole in the PO_4_^3−^ unit (POHC), the absorption bands located at 250 nm and 380 nm are ascribed to PO_4_^4−^ and PO_3_^2−^ defect centers, respectively, of which only POHC is a hole defect [28,29]. The absorption intensity of the Nd-doped phosphate glasses in the range of 250–600 nm is enhanced as the radiation dose increases, as shown in Figure 1b. The unirradiated Nd-doped phosphate glasses produce absorption at 360, 524, and 582 nm, corresponding to the Nd^3+^ ions energy level transition of ^4^I_9/2_ → (^4^G_3/2_ + ^4^G_5/2_ + ^2^I_11/2_ + ^4^G_1/2_), (^4^G_9/2_ + ^4^G_7/2_ + ^2^K_13/2_), (^4^G_5/2_ + ^2^G_7/2_), respectively [30], which does not change significantly as the radiation dose increases. The irradiated Ag-doped glasses generate an absorption peak with a central wavelength of 310 nm, which indicates that the irradiated luminous center can absorb UV light, and as the radiation dose increases, the absorption intensity also gradually improves, as shown in Figure 1c. The unirradiated Ag–Nd-codoped phosphate glasses have absorption peaks at 360, 524, and 582 nm, corresponding to the Nd^3+^ ions absorption, as shown in Figure 1d. After irradiation, an absorption intensity is generated at 310 nm, which corresponds to the absorption of the Ag defect centers. The horizontal axis of the induced absorption spectrums of Ag-doped glass and Ag–Nd-codoped glass was changed to energy (eV), and Gaussian peak-fitting was performed at 250 Gy, as shown in Figure 1e,f. According to reports [13,14,16], the induced absorption spectrum of Ag-doped glass mainly consists of three absorption peaks. The optical absorption band at about 3.35 eV (370 nm) may be attributed to Ag^0^ ions, and the bands at about 3.87 eV (320 nm), 4.30 eV (288 nm) may be attributed to Ag^2+^ ions [13,14,16].

The Ag-doped phosphate glasses contain Ag^+^ ions and phosphate PO_4_^3−^. After irradiation, PO_4_^3−^ will lose an electron and form a positron trap hole (hPO_4_). At this time, the lost electrons of PO_4_^3−^ will bind Ag^+^ ions to change Ag^+^ into Ag^0^. Similarly, hPO_4_ will bind Ag^+^ ions to change Ag^+^ into Ag^2+^ [13,14,15,16,17]. Finally, Ag^0^ and Ag^2+^ ions can form stable luminescence centers at room temperature, and neither need other defects to compensate for the charge. Only when glass is annealed at 400 °C for one hour, can the luminescent center gain enough energy to return to the ground state of silver ions, as opposed to other materials [31]. In the meantime, the Ag ions of different valence states in glasses will also aggregate to form an Ag_m_^n+^ type center, where n and m are integers [17,32], Such as Ag^2+^ (Ag^0^ + Ag^+^). The RPL intensity of phosphate glasses is mainly affected by the rate of Ag^+^ trapped holes [9]. The 310 nm absorption intensities of Ag-doped and Ag–Nd-codoped phosphate glasses at the 200 Gy radiation dose with relative intensities of 0.52 and 0.78, respectively. The high absorption strength of Ag–Nd-codoped glasses indicates that Ag^+^ ions are more likely to recapture hole defects and form more luminescent centers.

### 3.2. The Fluorescence Spectra of the Ag-Doped and Ag–Nd-Codoped Phosphate Glasses at 310 nm Excitation

The RPL intensity of irradiated Ag-doped metaphosphate glass reached a maximum after one week, which heralds the completion of build-up at room temperature [19]. In this work, the interval between the sample radiation time and the test time is more than one week, so the data used in this paper was collected after the completion of the build-up. It is generally believed that RPL contains two emission peaks, namely: a weaker emission peak at 450 nm (blue) and a stronger emission peak at 650 nm (orange). The former is the defect luminescence of Ag^0^. However, there is no consensus on which type of Ag defect luminescence belongs to the latter. There are two primary viewpoints regarding this: one holds that the orange RPL is related to Ag^2+^ defect center luminescence [15,33,34], the other holds that it is related to the co-luminescence of Ag^2+^ and Ag_2_^+^ [1,32,35]. Unlike chalcogenide glasses [36,37], phosphate glasses have short chemical bonds and compact structures. Consequently, the effect of holes on radiation performance is not considered for the time being.

**Figure 2:**
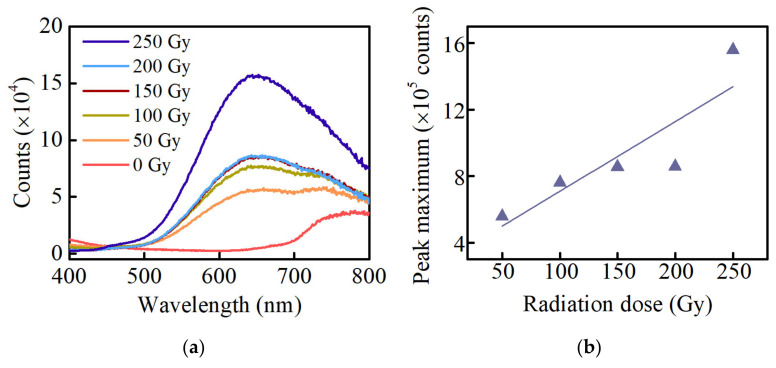
(**a**) The fluorescence spectra of the Ag-doped phosphate glasses at different radiation doses; (**b**) the linear relationship between the RPL peak maximum intensity of the Ag defect centers at 650 nm and the radiation dose in the Ag-doped phosphate glasses. (310 nm excitation).

Figure 2a shows the fluorescence spectra of Ag-doped phosphate glasses at different radiation doses (310 nm excitation). The irradiated glasses produce fluorescence peaks at 450 and 650 nm, and the peak intensity at 450 nm is very weak compared to 650 nm. The intensity of the orange RPL gradually rises with increasing radiation dose. However, the fluorescence peaks of 150 and 200 Gy-irradiated glasses almost coincide, which has been measured several times. Figure 2b shows the linear relationship between the RPL peak maximum intensity of the Ag defect centers at 650 nm and the radiation dose in the Ag-doped phosphate glasses. It can be seen from Figure 2b that the fluorescence responsivity of the RPL peak intensity to dose is poor.

**Figure 3:**
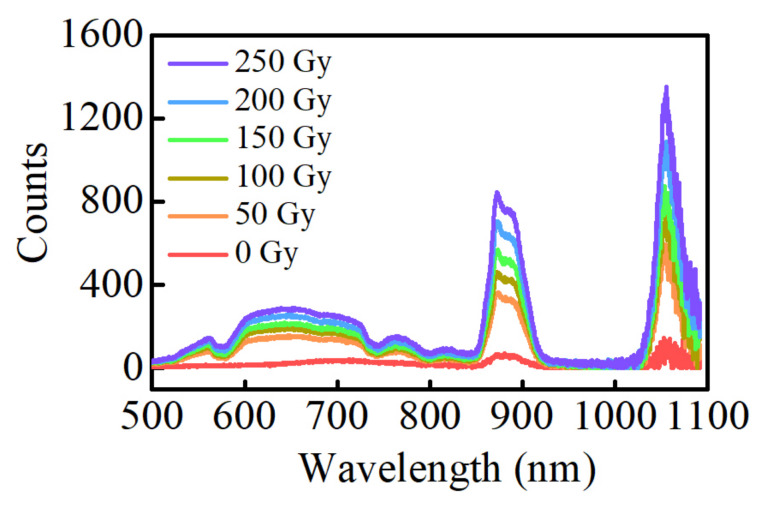
The fluorescence spectra of the Ag–Nd-codoped phosphate glasses at different radiation doses (310 nm excitation).

Figure 3 shows the relationship between the fluorescence spectrums of Ag–Nd-codoped phosphate glasses and the radiation dose at 310 nm excitation. The fluorescence spectrum of the unirradiated glasses show only small fluorescence peaks at 880 nm and 1054 nm, which is because the Nd^3+^ ions absorb excitation light at 310 nm, transitioning from the ground state to the ^2^H_9/2_ energy level, then to the ^4^F_3/2_ energy level without radiation, and finally from the excited state ^4^F_3/2_ to the ground state ^4^I_9/2_ and ^4^I_11/2_ [38]. The fluorescence spectrums of the irradiated glasses show broad fluorescence peaks in the range of 500–800 nm, fluorescence depressions at 524, 580, 684, 750, and 800 nm, and its intensity increases at 880 nm and 1054 nm. The broad fluorescence peak in the range of 500–800 nm is due to the RPL of the Ag defect centers. The depression is because the Nd^3+^ ions absorb the photons generated by the Ag defect centers, transitioning from the ground state ^4^I_9/2_ to the excited state ^4^G_5/2_ + ^4^G_7/2_ + ^2^K_13/2_, ^4^G_5/2_ + ^2^G_7/2_, ^4^F_9/2_, ^4^F_7/2_ + ^4^S_3/2_ and ^2^H_9/2_ + ^4^F_5/2_ [30], respectively. Both the RPL intensity of Ag defect centers and the fluorescence intensity of Nd^3+^ ions improve as the radiation dose increases, indicating that the number of Ag defect centers is rising and the energy transfer between Ag defect centers and Nd^3+^ ions is enhancing. While the fluorescence peak shapes of Ag defect centers and Nd^3+^ ions do not change with increasing radiation dose.

**Figure 4:**
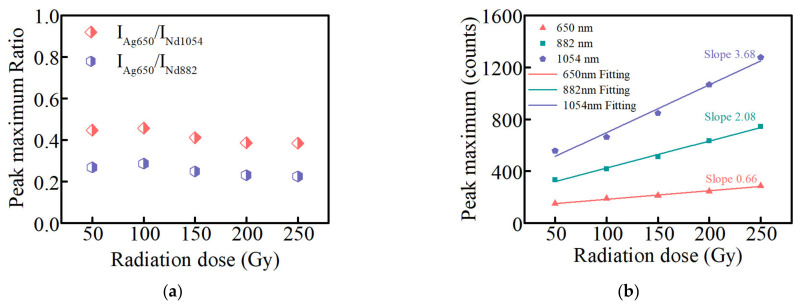
(**a**) the peak maximum ratio of Ag defect center at 650 nm to that of Nd^3+^ ions at 882 and 1054 nm at different radiation doses; (**b**) the linear relationship between the RPL peak maximum intensity of Ag defect centers at 650 nm and the fluorescence peak maximum intensity of Nd^3+^ ions at 882 and 1054 nm and the radiation dose in Ag–Nd-codoped phosphate glasses (310 nm excitation).

Figure 4a shows the peak maximum ratio of Ag defect center at 650 nm to that of Nd^3+^ ions at 882 and 1054 nm at different radiation doses (310 nm excitation). When the radiation dose of glasses is 50 Gy, the peak maximum ratios are 0.27 and 0.45, respectively. As the radiation dose increases, the ratios show a slight downward trend but are around 0.25 and 0.40, respectively. The slight decreasing trend may be attributed to the enhanced energy transfer from Ag defects to Nd^3+^ ions, but the effect of energy transfer enhancement is not obvious. Figure 4b shows the linear relationship between the RPL peak maximum intensity of the Ag defect centers at 650 nm and the fluorescence peak maximum intensity of Nd^3+^ ions at 882 and 1054 nm and the radiation dose in Ag–Nd-codoped phosphate glasses, plotted with red, green, and blue lines, respectively. The fluorescence response reflects the sensitivity of the glass dosimeter to radiation, and the greater the slope, the higher the sensitivity [8]. The RPL peak maximum intensity of the Ag defect centers and the fluorescence peak maximum intensity of the Nd^3+^ ions at 882 and 1054 nm with the radiation dose show good linear relationships, and the slopes are 0.66, 2.08, and 3.44, respectively. The slope of the RPL peak maximum intensity and radiation dose is taken as the standard values (the red line in Figure 4b), and the slope coefficients of the fluorescence peak maximum intensity of the Nd^3+^ ions at 882 and 1054 nm versus the radiation dose become 3.15 and 5.58, respectively. The good linear relationship and the improvement of the slope coefficient at 310 nm excitation indicate that the Ag–Nd-codoped phosphate glasses can be used as an RPL dosimeter.

### 3.3. The Infrared Transmission Spectra

**Figure 5:**
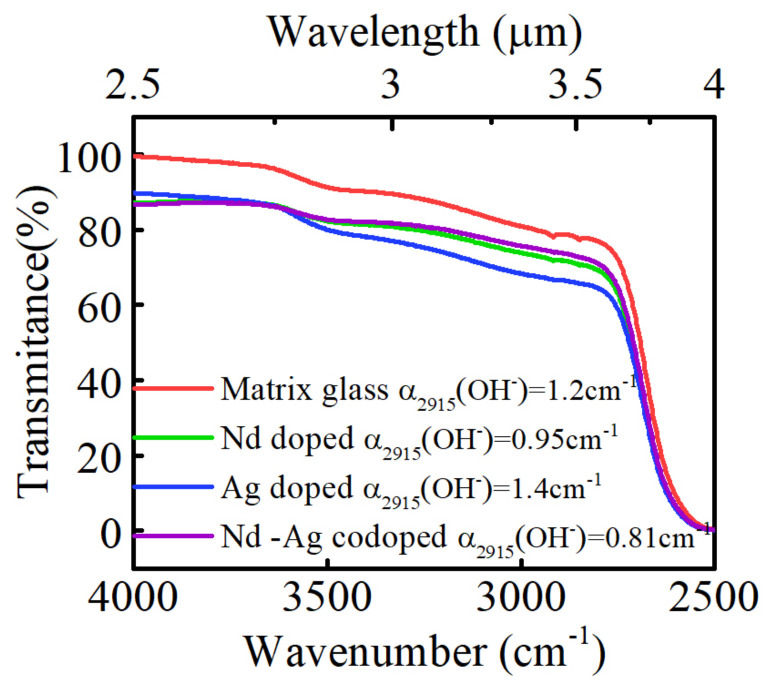
The infrared transmission spectra of unirradiated phosphate glass samples in the range of 2500–4000 cm^−1^ (4–2.5 μm).

Figure 5 reveals the infrared (IR) transmittance spectra of unirradiated phosphate glass samples. The distinct absorption peaks around 3500 cm^−1^ and 3000 cm^−1^ are caused by free hydroxyl OH^−^ vibration. The absorption coefficient of hydroxyl OH^−^ is determined by the equation α (OH^−^) = log (T_0_/T)/L, where T_0_ refers to the maximum infrared transmittance of the glass, T is the transmittance in the 3000 cm^−1^ (3.33 um), and L represents the thickness of the sample. A higher hydroxyl absorption coefficient enhances the nonradiative transitions of the upper energy level and affects the lifetime, while the hydroxyl absorption coefficients α (OH^−^) of four different doped phosphate glasses are 1.2, 0.95, 1.4, and 0.81 cm^−1^, respectively. Their values are lower than 1.5 cm^−1^, indicating that these four groups of glasses exhibit good water removal and the fluorescence lifetimes of Ag defect centers reflect the effect of the matrix.

### 3.4. The Fluorescence Lifetime at 310 nm Excitation

The RPL lifetime of the Ag defect centers at 650 nm consists of three components, namely, the background dose short lifetime τ_1_, the RPL lifetime τ_2_, and the background dose long lifetime τ_3_, generally τ_1_ < 1 us, τ_3_ > 20 us [39]. Background dose lifetimes τ_1_ and τ_3_ are independent of radiation, and originate from contamination and the internal defects of glasses, while τ_2_ represents the RPL lifetime of the Ag defect centers.

**Figure 6:**
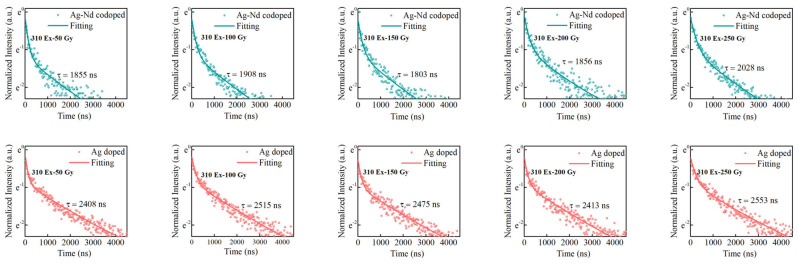
The RPL lifetime of Ag defect centers at 650 nm in Ag-doped and Ag–Nd-codoped phosphate glasses at different radiation doses (310 nm excitation).

To further reveal the existence of the energy transfer from Ag defect centers to Nd^3+^ ions under UV excitation in irradiated Ag–Nd-codoped phosphate glasses, the RPL lifetime of Ag defect centers at 650 nm in Ag-doped and Ag–Nd-codoped phosphate glasses were tested at different radiation doses, as shown in Figure 6. At a radiation dose of 50 Gy, the RPL fluorescence lifetimes τ_2_ of Ag-doped and Ag–Nd-codoped phosphates are 2408 and 1855 ns, respectively. Since the RPL of the Ag defect centers and the absorption spectra of the Nd^3+^ ions overlap in the range of 500–800 nm, energy can be transferred from the high-energy state of the Ag defect centers to the high-energy state of the Nd^3+^ ions. The number of excited state particles in the Ag defect centers then decreases rapidly, resulting in a decrease in the RPL lifetime τ_2_. The work of Ref. [39] investigated the fluorescence lifetimes of Ag defect centers at different radiation doses in Ag-doped metaphosphate glass, and it was found that the lifetime of Ag defect centers was independent of the radiation dose within the error range. In our work, the fluorescence lifetime of the Ag defect center shows no significant change with increasing radiation dose. Furthermore, we have removed water during glass melting to ensure that the non-radiative transition is not affected by the hydroxyl in the glass, as shown in Figure 5. The energy transfer efficiency is calculated according to the formula [40,41,42,43]:

(1) $$\eta =1-\frac{\tau_{\mathrm{Ag}–\mathrm{Nd}}}{\tau_{\mathrm{Ag}}}$$

which is commonly used in the fields of luminescence research and luminescent materials for energy transfer between the donor and the acceptor. Among them, τ_Ag_ and τ_Ag–Nd_ are the RPL lifetimes of Ag-doped and Ag–Nd-codoped glasses, respectively, and η represents the energy transfer from Ag defect centers to Nd^3+^ ions. The values of background dose lifetime τ_1_, RPL lifetime τ_2_ (τ_A__g_, τ_Ag–Nd_), and energy transfer efficiency η at different radiation doses are shown in Table 2. At the doses of 50, 100, 150, 200, and 250 Gy, the energy transfer efficiencies are 22.97%, 24.13%, 27.15%, 23.08%, and 20.56%, respectively. The data varied by 25%, demonstrating that the energy transfer efficiency η from Ag defect centers to Nd^3+^ ions does not significantly change with radiation dose.

### 3.5. The Fluorescence Spectra of the Ag–Nd-Codoped Phosphate Glasses at 380 nm

**Figure 7:**
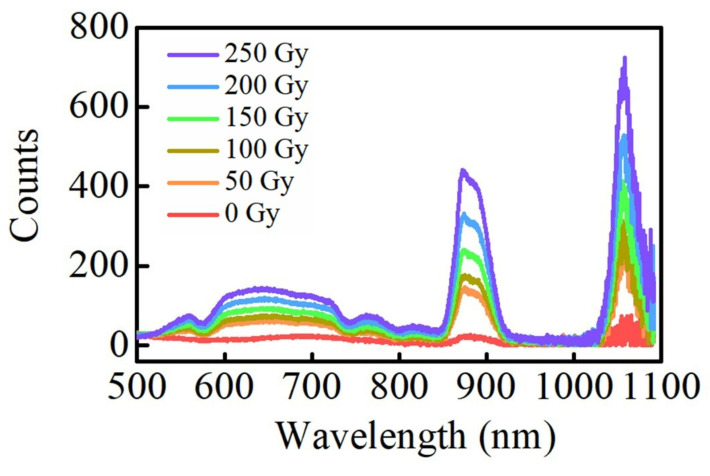
The fluorescence spectra of the Ag–Nd-codoped phosphate glasses at different radiation doses (380 nm excitation).

Due to the absorption of 310 nm excitation light by Nd^3+^ ions, the value of energy transfer η will cause deviations. From the absorption spectrum of Nd-doped phosphate glasses, Nd^3+^ ions do not absorb excitation light in the range of 370–420 nm. Therefore, the fluorescence spectra and lifetimes of Ag–Nd-codoped glasses were measured at 380 nm excitation, as shown in Figure 7. For the unirradiated glass, Nd^3+^ ions still have very low-intensity oscillation peaks at 880 and 1054 nm, which may be attributed to energy transfer caused by other defects or background noise. The fluorescence spectra of irradiated glasses show broad fluorescence peaks in the range of 500–800 nm, collapses at 524, 580, 684, 750, and 800 nm, with their intensity increasing at 880 nm and 1054 nm. The fluorescence peak shapes of Ag defect centers and Nd^3+^ ions do not change with the increase in radiation dose. The fluorescence spectra rule for 380 nm excitation is comparable to that for 310 nm excitation, and the relevant description has been provided above. Compared with 310 nm, the fluorescence peak intensities of RPL at 650 nm and Nd^3+^ ions at 880 and 1054 nm both decreased at 380 nm excitation. The former is attributed to the fact that 380 nm is not the optimal absorption wavelength of Ag defect centers, while the latter is due to the reduced number of photons from Ag defect centers to Nd^3+^ ions.

**Figure 8:**
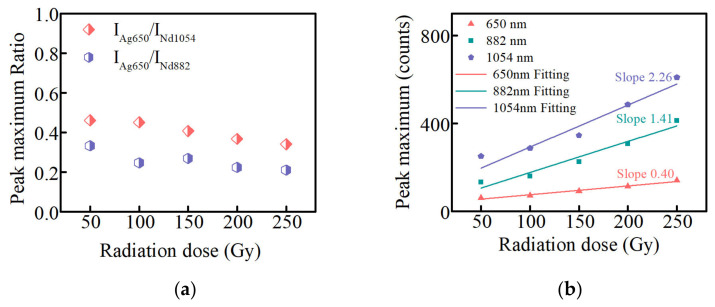
(**a**) the peak maximum ratio of the Ag defect center at 650 nm compared to that of Nd^3+^ ions at 882 and 1054 nm at different radiation doses; (**b**) the linear relationship between the RPL peak maximum intensity of Ag defect centers at 650 nm and the fluorescence peak maximum intensity of Nd^3+^ ions at 882 and 1054 nm and the radiation dose in Ag–Nd-codoped phosphate glasses (380 nm excitation).

Figure 8a shows that the peak maximum ratio of the Ag defect center lies at 650 nm, compared to that of Nd^3+^ ions at 882 and 1054 nm at different radiation doses at 380 nm excitation. When the radiation dose of glasses is 50 Gy, the fluorescence intensity ratios are 0.33 and 0.46, respectively. There is a slight decreasing trend with increasing radiation dose, but the ratios are around 0.25 and 0.40, respectively. Figure 8b shows the linear relationship between the RPL peak maximum intensity of the Ag defect centers at 650 nm and the fluorescence peak maximum intensity of Nd^3+^ ions at 882 and 1054 nm and the radiation dose, plotted with red, green, and blue lines, respectively. The RPL peak maximum intensity of the Ag defect centers and the fluorescence peak maximum intensity of Nd^3+^ ions at 882 and 1054 nm present a good linear relationship with the radiation dose, and the slopes are 0.40, 1.41, and 2.26 respectively. The slope of RPL intensity and radiation dose are taken as the standard values (the red line in Figure 8b), and the slope coefficients of the fluorescence intensity of the Nd^3+^ ions at 882 and 1054 nm versus the radiation dose become 3.53 and 5.65, respectively. The good linear relationship and the improvement of the slope coefficient at 380 nm excitation indicate that the Ag–Nd-codoped phosphate glasses can be used as an RPL dosimeter.

### 3.6. The Fluorescence Lifetime at 380 nm Exciatition

**Figure 9:**
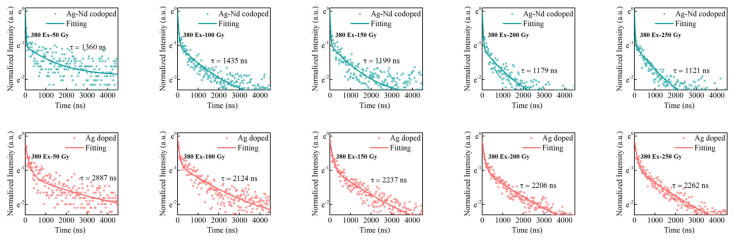
The RPL lifetime of Ag defect centers at 650 nm in Ag-doped and Ag–Nd-codoped phosphate glasses at different radiation doses (380 nm excitation).

To further reveal the energy transfer efficiency of Ag defect centers to Nd^3+^ ions at 380 nm excitation, the RPL lifetime of Ag defect centers at 650 nm in Ag-doped and Ag–Nd-codoped phosphate glasses were tested at different radiation doses, as shown in Figure 9. The values of background dose lifetime τ_1_, RPL lifetime τ_2_, and energy transfer efficiency η at different radiation doses are demonstrated in Table 3. The energy transfer efficiencies are 52.89%, 32.43%, 46.40%, 46.55%, and 49.11%, respectively, at the doses of 50, 100, 150, 200, and 250 Gy. Except for 100 Gy, the energy transfer efficiency is stable at around 50%. The reason why 380 nm excitation has higher energy transfer efficiency is that Nd^3+^ ions do not absorb 380 nm excitation light, resulting in the energy of the Nd^3+^ ions transition coming entirely from the RPL of the Ag defect centers, so the number of excited state particles in the Ag defect centers decreases rapidly.

The 380 nm excitation has the following advantages over 310 nm: (1) greater energy transfer efficiency η from Ag defect centers to Nd^3+^ ions, and more accurate calculation of η; (2) larger slope coefficient and higher sensitivity. Taking the slope of RPL intensity and radiation dose as the standard value, the slope coefficients of the fluorescence intensity of the Nd^3+^ ions at 882 and 1054 nm and the radiation dose are 3.53 and 5.65, respectively, while they are 3.15 and 5.58 at 310 nm.

## 4. Conclusions

In contrast to the traditional Ag-single-doped glasses used for RPL glass dosimeters, this work developed and studied an Ag–Nd-codoped phosphate glass material with 99.5 (0.7LiPO_3_–0.3Al (PO_3_)_3_)–0.25Ag_2_O–0.25Nd_2_O_3_. After irradiating, the Ag defect center can absorb UV light of 280–400 nm and emit 650 nm fluorescence in the range of 500–800 nm. Flourescence depressions at 524, 580, 684, 750, and 800 nm of the Ag defect center, and fluorescence increases of 880 nm and 1054 nm of Nd^3+^ ions are demonstrated. These two facts indicate that there is an energy transfer from the Ag defect center to Nd^3+^ ions. The existence of the energy transfer is further confirmed by the fluorescence lifetime of the Ag defect center at 650 nm, which is decreased greatly after Nd codoping. While using 310 nm excitation for Ag–Nd-codoped glass, the RPL peak maximum intensity of the Ag defect centers and the fluorescence peak maximum intensity of Nd^3+^ ions at 882 and 1054 nm present a good linear relationship with the radiation dose, and present slopes of 0.66, 2.08, and 3.44, respectively. While using 380 nm excitation, these three fluorescence peak maximum intensities also increase linearly, presenting slopes of 0.40, 1.41, and 2.26, respectively. Notably, it is determined that the energy transfer efficiency at 310 nm and 380 nm excitation is about 25% and 50%, respectively. Therefore, the newly developed Ag–Nd-codoped phosphate glass should have a higher RPL fluorescence response because of the higher energy transfer efficiency under 380 nm excitation, and also have a higher RPL detection sensitivity because of the higher linear slopes under 310 nm excitation.

In general, the new Ag–Nd-codoped phosphate glass we prepared and developed can be applied to new RPL glass sensors and dosimeters, as it is proven to be a multi-wavelength RPL fluorescence glass material.

## Figures and Tables

**Table 1:** Four Differently Doped Phosphate Glass Compositions (mol%).

Sample	LiPO_3_	Al (PO_3_)_3_	Nd_2_O_3_	Ag_2_O
Matrix glass	70	30	−	−
Nd-doped	69.825	29.925	0.25	−
Ag-doped	69.825	29.925	−	0.25
Ag–Nd-codoped	69.65	29.85	0.25	0.25

**Table 2:** Background doses lifetime τ_1_ and RPL lifetime τ_2_ of Ag coped and Ag–Nd-codoped phosphate glasses; energy transfer efficiency η from Ag defect centers to Nd^3+^ ions in Ag–Nd-codoped glasses at different radiation doses (310 nm excitation).

Radiation Doses	Type of Phosphate Glasses	Background Doses Lifetime τ_1_, RPL Lifetime τ_2_ (the Error Is Less Than 5%)	Energy Transfer Efficiency η
50 Gy	Ag-doped	τ_1_ = 116 ns, τ_2_ = 2408 ns	22.97%
	Ag–Nd-codoped	τ_1_ = 80 ns, τ_2_ = 1855 ns	
100 Gy	Ag-doped	τ_1_ = 169 ns, τ_2_ = 2515 ns	24.13%
	Ag–Nd-codoped	τ_1_ = 155 ns, τ_2_ = 1908 ns	
150 Gy	Ag-doped	τ_1_ = 138 ns, τ_2_ = 2475 ns	27.15%
	Ag–Nd-codoped	τ_1_ = 161 ns, τ_2_ = 1803 ns	
200 Gy	Ag-doped	τ_1_ = 143 ns, τ_2_ = 2413 ns	23.08%
	Ag–Nd-codoped	τ_1_ = 194 ns, τ_2_ = 1856 ns	
250 Gy	Ag-doped	τ_1_ = 224 ns, τ_2_ = 2553 ns	20.56%
	Ag–Nd-codoped	τ_1_ = 224 ns, τ_2_ = 2028 ns	

**Table 3:** Background doses lifetime τ_1_ and RPL lifetime τ_2_ of Ag-doped and Ag–Nd-codoped phosphate glasses; energy transfer efficiency η from Ag defect centers to Nd^3+^ ions in Ag–Nd-codoped glasses at different radiation doses (380 nm excitation).

Radiation Doses	Type of Phosphate Glasses	Background Doses Lifetime τ_1_, RPL Lifetime τ_2_ (the Error Is Less Than 5%)	Energy Transfer Efficiency η
50 Gy	Ag-doped	τ_1_ = 179 ns, τ_2_ = 2887 ns	52.89%
	Ag–Nd-codoped	τ_1_ = 26 ns, τ_2_ = 1360 ns	
100 Gy	Ag-doped	τ_1_ = 92 ns, τ_2_ = 2124 ns	32.43%
	Ag–Nd-codoped	τ_1_ = 52 ns, τ_2_ = 1435 ns	
150 Gy	Ag-doped	τ_1_ = 135 ns, τ_2_ = 2237 ns	46.40%
	Ag–Nd-codoped	τ_1_ = 30 ns, τ_2_ = 1199 ns	
200 Gy	Ag-doped	τ_1_ = 122 ns, τ_2_ = 2206 ns	46.55%
	Ag–Nd-codoped	τ_1_ = 41 ns, τ_2_ = 1179 ns	
250 Gy	Ag-doped	τ_1_ = 137 ns, τ_2_ = 2262 ns	49.11%
	Ag–Nd-codoped	τ_1_ = 23 ns, τ_2_ = 1121 ns	

## Data Availability

The data that support the findings of this study are contained within the article.
